# Burden of diarrhea in the Eastern Mediterranean Region, 1990–2015: Findings from the Global Burden of Disease 2015 study

**DOI:** 10.1007/s00038-017-1008-z

**Published:** 2017-08-03

**Authors:** GBD 2015 Eastern Mediterranean Region Diarrhea Collaborators, Ibrahim Khalil, Charbel El Bcheraoui, Raghid Charara, Maziar Moradi-Lakeh, Ashkan Afshin, Nicholas J. Kassebaum, Michael Collison, Adrienne Chew, Kristopher J. Krohn, Farah Daoud, Danny Colombara, Kyle J. Foreman, William W. Godwin, Michael Kutz, Mojde Mirarefin, Puja C. Rao, Robert Reiner, Christopher Troeger, Haidong Wang, Haftom Niguse Abraha, Remon Abu-Elyazeed, Laith J. Abu-Raddad, Aliasghar Ahmad Kiadaliri, Alireza Ahmadi, Muktar Beshir Ahmed, Khurshid Alam, Reza Alizadeh-Navaei, Rajaa Al-Raddadi, Khalid A. Altirkawi, Nelson Alvis-Guzman, Nahla Anber, Palwasha Anwari, Tesfay Mehari Atey, Euripide Frinel G. Arthur Avokpaho, Umar Bacha, Shahrzad Bazargan-Hejazi, Neeraj Bedi, Isabela M. Bensenor, Adugnaw Berhane, Pascal Obong Bessong, Addisu Shunu Beyene, Zulfiqar A. Bhutta, Geoffrey Colin Buckle, Zahid A. Butt, Hadi Danawi, Amare Deribew, Shirin Djalalinia, Manisha Dubey, Aman Yesuf Endries, Babak Eshrati, Seyed-Mohammad Fereshtehnejad, Florian Fischer, Tsegaye Tewelde Gebrehiwot, Harish Chander Gugnani, Randah Ribhi Hamadeh, Samer Hamidi, Abdullatif Husseini, Spencer Lewis James, Jost B. Jonas, Yousef Saleh Khader, Ejaz Ahmad Khan, Gulfaraz Khan, Jagdish Khubchandani, Niranjan Kissoon, Jacek A. Kopec, Ai Koyanagi, Barthelemy Kuate Defo, Heidi J. Larson, Asma Abdul Latif, Raimundas Lunevicius, Hassan Magdy Abd El Razek, Mohammed Magdy Abd El Razek, Reza Majdzadeh, Azeem Majeed, Reza Malekzadeh, Peter Memiah, Ziad A. Memish, Walter Mendoza, Desalegn Tadese Mengistu, Shafiu Mohammed, Srinivas Murthy, Josephine Wanjiku Ngunjiri, Felix Akpojene Ogbo, Farshad Pourmalek, Mostafa Qorbani, Amir Radfar, Anwar Rafay, Vafa Rahimi-Movaghar, Rajesh Kumar Rai, Usha Ram, David Laith Rawaf, Salman Rawaf, Andre M. N. Renzaho, Satar Rezaei, Gholamreza Roshandel, Mahdi Safdarian, Mohammad Ali Sahraian, Payman Salamati, Abdallah M. Samy, Juan Ramon Sanabria, Benn Sartorius, Sadaf G. Sepanlou, Masood Ali Shaikh, Mika Shigematsu, Badr H A. Sobaih, Chandrashekhar T. Sreeramareddy, Bryan L. Sykes, Arash Tehrani-Banihashemi, Mohamad-Hani Temsah, Abdullah Sulieman Terkawi, Tenaw Yimer Tiruye, Roman Topor-Madry, Kingsley Nnanna Ukwaja, Stein Emil Vollset, Tolassa Wakayo, Andrea Werdecker, Charles Shey Wiysonge, Abdulhalik Workicho, Mohsen Yaghoubi, Mehdi Yaseri, Muluken Yenesew, Naohiro Yonemoto, Mustafa Z. Younis, Maysaa El Sayed Zaki, Bassel Zein, Sanjay Zodpey, Aisha O. Jumaan, Theo Vos, Simon I. Hay, Mohsen Naghavi, Christopher J L. Murray, Ali H. Mokdad

**Affiliations:** Institute for Health Metrics and Evaluation, 2301 5th Ave, Suite 600, Seattle, WA 98121 USA

**Keywords:** Eastern Mediterranean Region, Burden of disease, Diarrheal diseases

## Abstract

**Objectives:**

Diarrheal diseases (DD) are an important cause of disease burden, especially in children in low-income settings. DD can also impact children’s potential livelihood through growth faltering, cognitive impairment, and other sequelae.

**Methods:**

As part of the Global Burden of Disease study, we estimated DD burden, and the burden attributable to specific risk factors and etiologies, in the Eastern Mediterranean Region (EMR) between 1990 and 2015. We calculated disability-adjusted life-years (DALYs)—the sum of years of life lost and years lived with disability—for both sexes and all ages.

**Results:**

We estimate that over 103,692 diarrhea deaths occurred in the EMR in 2015 (95% uncertainty interval: 87,018–124,692), and the mortality rate was 16.0 deaths per 100,000 persons (95% UI: 13.4–19.2). The majority of these deaths occurred in children under 5 (63.3%) (65,670 deaths, 95% UI: 53,640–79,486). DALYs per 100,000 ranged from 304 (95% UI 228–400) in Kuwait to 38,900 (95% UI 25,900–54,300) in Somalia.

**Conclusions:**

Our findings will guide evidence-based health policy decisions for interventions to achieve the ultimate goal of reducing the DD burden.

**Electronic supplementary material:**

The online version of this article (doi:10.1007/s00038-017-1008-z) contains supplementary material, which is available to authorized users.

## Introduction

Rigorous public health efforts resulted in a significant decline in mortality due to diarrheal diseases (DD) over the past 20 years. However, these diseases continue to cause a major global disease burden, especially in children under 5 years of age. In addition, the incidence of childhood diarrhea in low-income countries has not declined as rapidly as mortality (GBD 2015 Risk Factors Collaborators 2016). In the most recent Global Burden of Disease (GBD) study, DD was the fourth-leading cause of death among children under 5, responsible for 499,000 deaths (95% UI: 447,000–558,000), representing 8.6% of all deaths in this age group (GBD 2015 Risk Factors Collaborators 2016). For those who survive these illnesses and suffer from repeated infections by enteric pathogens during the critical early years of life; DD can lead to serious, lifelong health consequences such as environmental enteric dysfunction (EED), growth faltering, impaired cognitive development, and reduced immune response to infection and vaccinations (Guerrant et al. 2013).

DD pathogen etiologic contribution may vary depending on the study’s geographic location, duration, or the population sampled (Lindsay et al. 2015). These infections are believed to be different in the developing world compared to the developed world with regard to a number of features, including earlier age of onset, multiple repeated exposures, greater diversity of pathogens, nutritional status of the host, and a number of others, such as co-infection, diet, and genetics (Heidt et al. 2014).

The Eastern Mediterranean Region (EMR) is home to more than 500 million people, representing a diverse group of 22 countries. EMR countries have diverse historical backgrounds, political and social contexts, and fiscal and cultural influences on their health care systems. The region has wide variation in per capita gross national product (GNP) (The World Bank 2016), which has a major influence on overall health spending and results in substantial health inequities both within and across countries. During recent years DD prevention efforts that focus on vaccines in the short term and improvements in water, sanitation, and hygiene in the long term have been impeded by warfare and political unrest in the region. These conflicts and wars also resulted in a huge problem of internal displacement and refugees (Mokdad et al. 2016).

Many countries in the EMR achieved important successes in the fight against DD in the 1970s and 1980s with the support of United Nations International Children’s Emergency Fund (UNICEF) and the World Health Organization (WHO) through the National Control of Diarrheal Diseases Project (NCDDP) (WHO 1992; Enzley et al. 1997). For example, Egypt’s program, which spanned from 1981 to 1991, was credited with significantly improving diarrheal case management (National Control Of Diarrheal Diseases Project 1988; El-Rafie et al. 1990; Cobb et al. 1996). However, over the last two decades, momentum has slowed (Forsberg et al. 2007). As of the date of this report, rotavirus vaccines have been introduced through National Immunization Programs in only nine countries in the region: Djibouti, Jordan, Libya, Morocco, Qatar, Saudi Arabia, Sudan, United Arab Emirates, and Yemen (PATH 2017).

In this report, we are updating our previous burden estimates (Khalil et al. 2016), pathogen distribution, and risk factors for diarrhea in children and adults in the EMR for 1990–2015.

## Methods

The Global Burden of Disease Study (GBD 2015) is a systematic, comprehensive effort to quantify health loss from more than 300 diseases and injuries, including diarrheal diseases and associated risk factors. The GBD estimation strategy, including for diarrheal diseases, has been described in detail elsewhere (Foreman et al. 2012; Flaxman et al. 2015). The burden of diarrheal diseases is measured in deaths, incidence, and disability-adjusted life-years (DALYs), which are the sum of years of life lost (YLLs) and years lived with disability (YLDs). The etiological burden was also estimated for 13 pathogens associated with diarrhea.

All estimates are produced by year and by age, for both sexes, and for all countries. In accordance with the guidelines for accurate and transparent health estimates reporting (GATHER), code for each step of the estimation process is available online on GitHub (http://www.ghdx.healthdata.org/gbd-2015-code) (Institute for Health Metrics and Evaluation). The methods of each of these steps are summarized below.

### Study region

The EMR countries were grouped according to per capita gross national income (GNI) into low-income countries (LICs) [Islamic Republic of Afghanistan (Afghanistan), Djibouti, Somalia, Republic of Yemen (Yemen)]; middle-income countries (MICs) [Arab Republic of Egypt (Egypt), Islamic republic of Iran (Iran), Iraq, Jordan, Lebanon, Libya, Morocco, Pakistan, Palestine, Sudan, Syrian Arab Republic (Syria), Tunisia]; and high-income countries (HICs) [Bahrain, Kuwait, Oman, Qatar, Saudi Arabia, and the United Arab Emirates (UAE)]. We defined LICs as those having a per capita GNI of $1045 or less, MICs as those with a per capita GNI between $1046 and $12,735, and HICs as countries with per capita GNI of $12,736 or greater (The World Bank 2016).

### Mortality

Cause-specific mortality estimates for diarrheal diseases were modeled using a Bayesian ensemble modeling process (GBD 2015 Mortality and Causes of Death Collaborators 2016). Diarrhea mortality data included vital registration and verbal autopsy sources. The modeling process estimated the mortality rate due to diarrhea for both sexes from 1990 to 2015 for all age groups in every country and subnational regions in select countries. We considered the following covariates: education, lag-dependent income, underweight, latitude, population density, improved water and sanitation sources, diarrhea risk factors summary, Socio-demographic Index (SDI), and rotavirus vaccine coverage. The ensemble model approach allows for a suite of models, weighted by out-of-sample predictive validity, to inform the final estimates.

### Morbidity

Diarrheal cases were defined as three or more loose stools in a 24-h period. As with mortality, morbidity was modeled at every year, sex, age, and geographic location in GBD 2015. The morbidity model used DisMod-MR 2.1, a Bayesian meta-analytic, age-integrating, mixed-effects model which is available online on EpiViz (http://www.vizhub/healthdata.org/epi) (Kassebaum et al. 2016). Diarrhea prevalence and incidence data from a systematic literature review, population-representative surveys, and hospital and health care utilization data informed the non-fatal model.

### Etiologies

Diarrhea cases and deaths were attributed to pathogens using a counterfactual approach that accounts for exposure to pathogens and for the association between each pathogen and diarrhea. A systematic literature review on the proportion of diarrhea cases that test positive for a set of pathogens was updated for GBD 2015. These data were used in the DisMod-MR framework to estimate the age, sex, year, and geographic distribution of pathogens in diarrheal episodes. The population attributable fraction (PAF) was used to identify the fraction of diarrhea cases and deaths due to each pathogen. The PAF was calculated as: (GBD 2015 Mortality and Causes of Death Collaborators 2016).

$${\text{PAF}} = {\text{Proportion}} \times \left( {1 - \frac{1}{\text{OR}}} \right),$$

where Proportion is the proportion of cases positive for a pathogen and the odds ratio (OR) is the odds of diarrhea given pathogen detection. The odds ratios were from a systematic reanalysis of the Global Enteric Multicenter Study (GEMS) (Kotloff et al. 2013), a multi-site case–control study of moderate-to-severe diarrhea in children under 5 that systematically tested nearly half of the original GEMS samples using a molecular quantitative polymerase chain reaction (qPCR) diagnostic (Liu et al. 2016). A mixed-effects conditional logistic regression model estimated the odds ratios for diarrhea including random effects on site to account for geographic variation.

Since the odds of diarrhea given pathogen presence were calculated using the qPCR diagnostic, we adjusted our proportion estimates to be comparable to qPCR-based estimates. The sensitivity and specificity of the non-molecular diagnostic techniques from GEMS were evaluated compared to the qPCR diagnostic, and these values were used to make this adjustment with this formula (Wickham 2009):

$${\text{Proportion}}_{\text{True}} = \frac{{({\text{Proportion}}_{\text{Observed}} \times {\text{Specificity}} - 1)}}{{({\text{Sensitivity}} + {\text{Specificity}} - 1)}}.$$

*Vibrio cholerae* and *Clostridium difficile* were estimated separately from the other pathogens in GBD. Cholera cases were estimated using proportion data from published studies to calculate an expected number of annual cases for each country and year, and those estimates were compared to the World Health Organization case notification data (WHO 2016) to estimate underreporting of cholera. Cholera deaths were estimated using case fatality data in DisMod-MR. Since *C. difficile* is frequently associated with hospital and health care utilization, hospital incidence data were modeled in DisMod-MR using hospital and health care utilization data with ICD codes for *C. difficile*.

All etiologies were estimated independently and for each year, geography, age, and sex.

### Risk factors

We also assessed diarrheal DALYs, YLLs, and YLDs attributable to childhood stunting, suboptimal breastfeeding, vitamin A deficiency, zinc deficiency, and water, sanitation, and hygiene (WASH). Risk factor attribution follows a general counterfactual approach where the exposure and relative risk of diarrhea were used to estimate the burden of the aforementioned risk factors (GBD 2015 Risk Factors Collaborators 2016).

### Socio-demographic Index

We evaluate associations between diarrhea and socio-demographic status using the Socio-demographic Index (SDI). The SDI is a composite measure developed for GBD 2015 that accounts for fertility rate, lag-dependent income per capita, and education (GBD 2015 Mortality and Causes of Death Collaborators 2016). To capture the average relationships for each age–sex group, we applied a simple least squares spline regression of the diarrhea mortality rate on SDI. The predicted diarrhea mortality rates from this regression were used as expected mortality rates based on SDI. The SDI is scaled from 0 to 1 where 0 represents the lowest possible observed SDI and 1 is the highest. SDI in 2015 in the EMR ranged from 0.27 in Somalia to 0.83 in Kuwait.

## Results

**Fig. 1:**
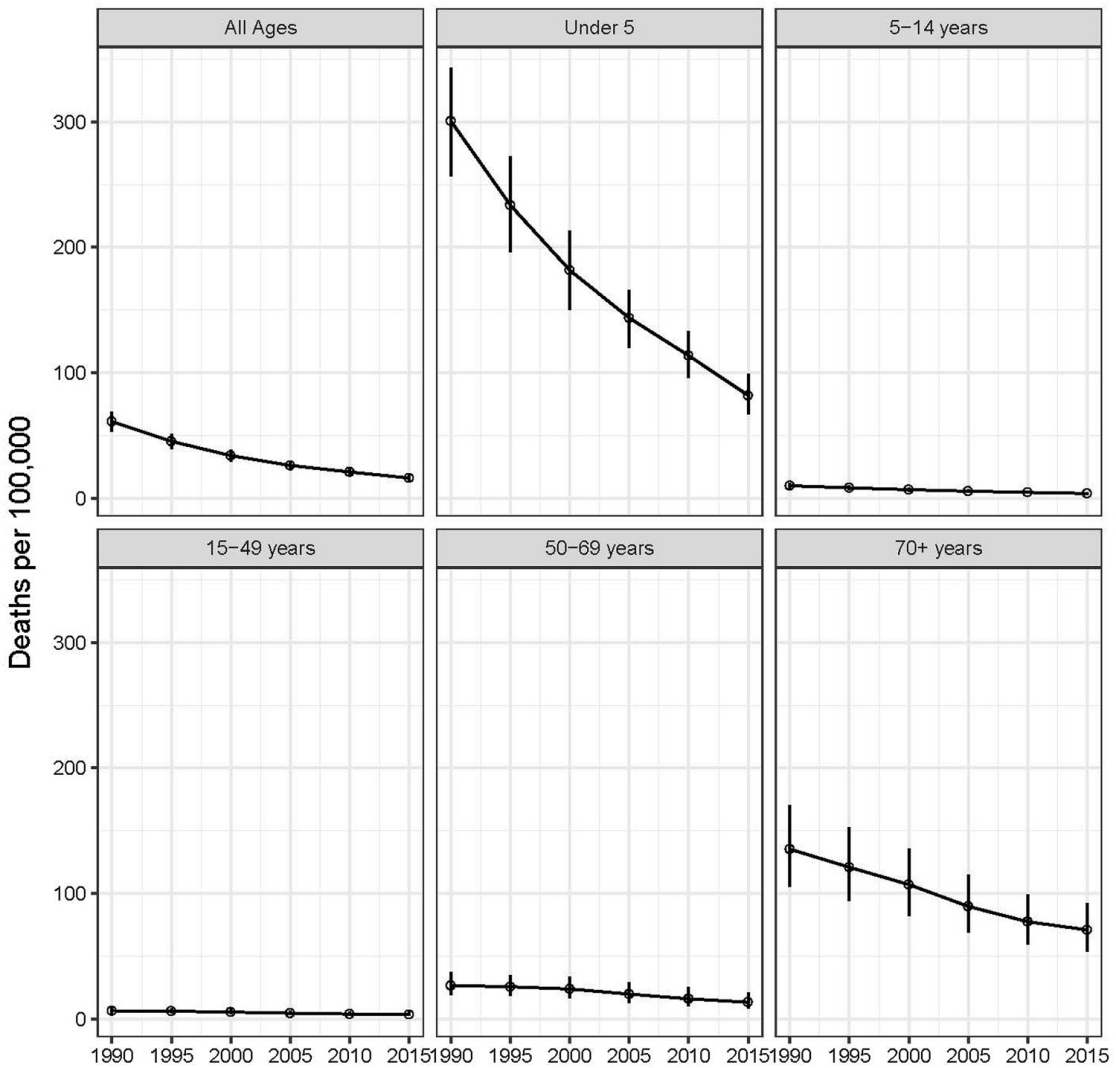
Diarrhea mortality rate per 100,000 population, by age, 1990–2015. The mortality rate in the Eastern Mediterranean Region is shown for six age groups over time from 1990 to 2015. Points represent mean estimates and error bars represent 95% uncertainty intervals (Global Burden of Disease 2015 study, Eastern Mediterranean Region, 1990–2015)

There were 103,692 diarrhea deaths in the EMR in 2015 (95% uncertainty interval (UI): 87,018–124,692), and the mortality rate was 16.0 deaths per 100,000 persons (95% UI: 13.4–19.2). The majority of these deaths occurred in children under 5 years old (63.3%) (65,670 deaths, 95% UI: 53,640–79,486). Although the greatest number of diarrhea deaths occurred in children under 5, diarrhea mortality was also high in the 70+ year age group. In fact, of the 22 countries in the EMR, the mortality rate was higher in the 70+ age group than the under-5 age group in 17 countries. Diarrhea mortality in the elderly was highest in Somalia (1695 per 100,000, 95% UI: 709–2896), followed by Djibouti and Pakistan (Table 1). Diarrhea mortality decreased over time in the EMR (Table 2; Fig. 1). Between 1990 and 2015, the number of diarrhea deaths decreased by 54% among all ages (95% UI: 43–62%) and 65% among children under 5 (95% UI: 55–73%). The fastest rate of decrease among children under 5 occurred in Iran (97%, 95% UI: 93–99%) and Syria (97%, 95% UI: 94–99%) and the slowest occurred in Somalia (11%, 95% UI: −40 to 45%) and Qatar (33%, 95% UI: −65 to 72%).

**Table 1:** The number of diarrhea deaths and mortality rate (per 100,000) in 2015 in the Eastern Mediterranean Region for each age group and sex (Global Burden of Disease 2015 study, Eastern Mediterranean Region, 2015)

Age	Sex	Deaths	Rate
All ages	Both	103,691.7 (87,018.3–124,692.2)	15.99 (13.42–19.23)
All ages	Female	50,679.8 (40,407.1–63,194.3)	16.14 (12.87–20.12)
All ages	Male	53,011.9 (42,617.5–65,304.5)	15.86 (12.75–19.53)
Under 5	Both	65,670.3 (53,639.7–79,485.9)	81.82 (66.83–99.04)
Under 5	Female	34,729.4 (26,435.3–45,011.1)	89.09 (67.81–115.46)
Under 5	Male	30,940.9 (23,892.9–39,342.5)	74.96 (57.89–95.32)
5–14 years	Both	4986.1 (3724.4–6501.8)	3.67 (2.74–4.78)
5–14 years	Female	2540.1 (1839.4–3414.2)	3.84 (2.78–5.16)
5–14 years	Male	2446 (1623.7–3486.2)	3.5 (2.32–4.99)
15–49 years	Both	11,546.4 (7917.3–18,792.8)	3.36 (2.3–5.47)
15–49 years	Female	4827.5 (3205.6–8471.4)	2.93 (1.94–5.13)
15–49 years	Male	6718.9 (4339.3–10,599.2)	3.76 (2.43–5.94)
50–69 years	Both	9457.6 (6095.2–15,147.7)	13.21 (8.51–21.16)
50–69 years	Female	3836.5 (2060–6875.3)	10.93 (5.87–19.58)
50–69 years	Male	5621.1 (3578.4–8787.7)	15.41 (9.81–24.08)
70+ years	Both	12,031.2 (9097.8–15,593.3)	70.83 (53.56–91.8)
70+ years	Female	4746.3 (3404.3–6304.8)	53.74 (38.55–71.39)
70+ years	Male	7285 (5054.7–10,119.4)	89.34 (61.99–124.1)

**Table 2:** The number of deaths, disability-adjusted life-years (DALYs), and episodes of diarrhea in the Eastern Mediterranean Region and the diarrhea mortality (per 100,000) and incidence (per person-year) rates among under-5 and all ages in 1990, 2000, and 2015 (Global Burden of Disease 2015 study, Eastern Mediterranean Region, 1990–2015)

Year	Age	Deaths		DALYs	Cases	
		Number	Rate	Number	Number	Rate
1990	Under 5	185,910 (127,308–243,615)	150.3 (102.9–196.9)	16,345,715 (11,335,370–21,282,470)	165,987,520 (146,655,489–187,730,433)	1.34 (1.19–1.52)
2000	Under 5	117,162 (76,746–160,181)	90.8 (59.5–124.1)	10,439,937 (6,959,185–14,117,350)	159,618,414 (140,515,118–181,990,713)	1.24 (1.09–1.41)
2015	Under 5	65,670 (42,393–95,304)	40.9 (26.4–59.4)	6,058,681 (4,045,101–8,618,353)	171,316,814 (152,549,199–193,090,921)	1.07 (0.95–1.20)
1990	All ages	225,746 (162,335–289,224)	30.5 (21.9–39.1)	18,258,180 (13,023,737–23,353,030)	244,994,495 (225,000,870–266,982,282)	0.33 (0.30–0.36)
2000	All ages	159,795 (113,345–210,958)	16.9 (12–22.4)	12,429,100 (8,755,478–16,336,533)	267,079,653 (247,580,591–290,033,675)	0.28 (0.26–0.31)
2015	All ages	103,692 (73,304–145,718)	8 (5.7–11.2)	7,816,119 (5,569,985–10,745,255)	318,037,078 (298,328,603–340,822,655)	0.25 (0.23–0.26)

**Fig. 2:**
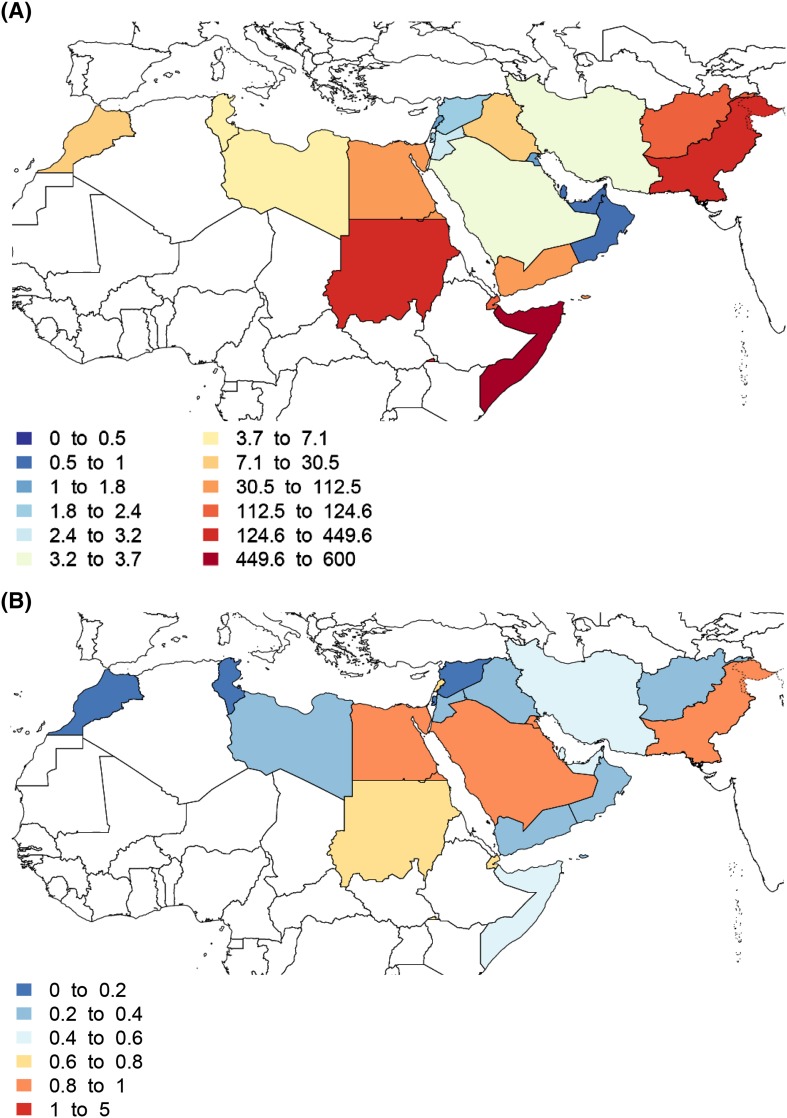
Diarrhea mortality among children under 5 in the Eastern Mediterranean Region, 2015. **a** The diarrhea mortality rate per 100,000 among children under 5 in 2015. **b** Ratio of the observed under 5 diarrhea mortality rate in 2015 to the expected mortality rate based on the Socio-demographic Index only. Values below 1 indicate that the diarrhea mortality rate is lower than would be expected based on the global relationship between mortality and SDI, and values above 1 indicate higher mortality rates than would be expected (Global Burden of Disease 2015 study, Eastern Mediterranean Countries, 2015)

**Fig. 3:**
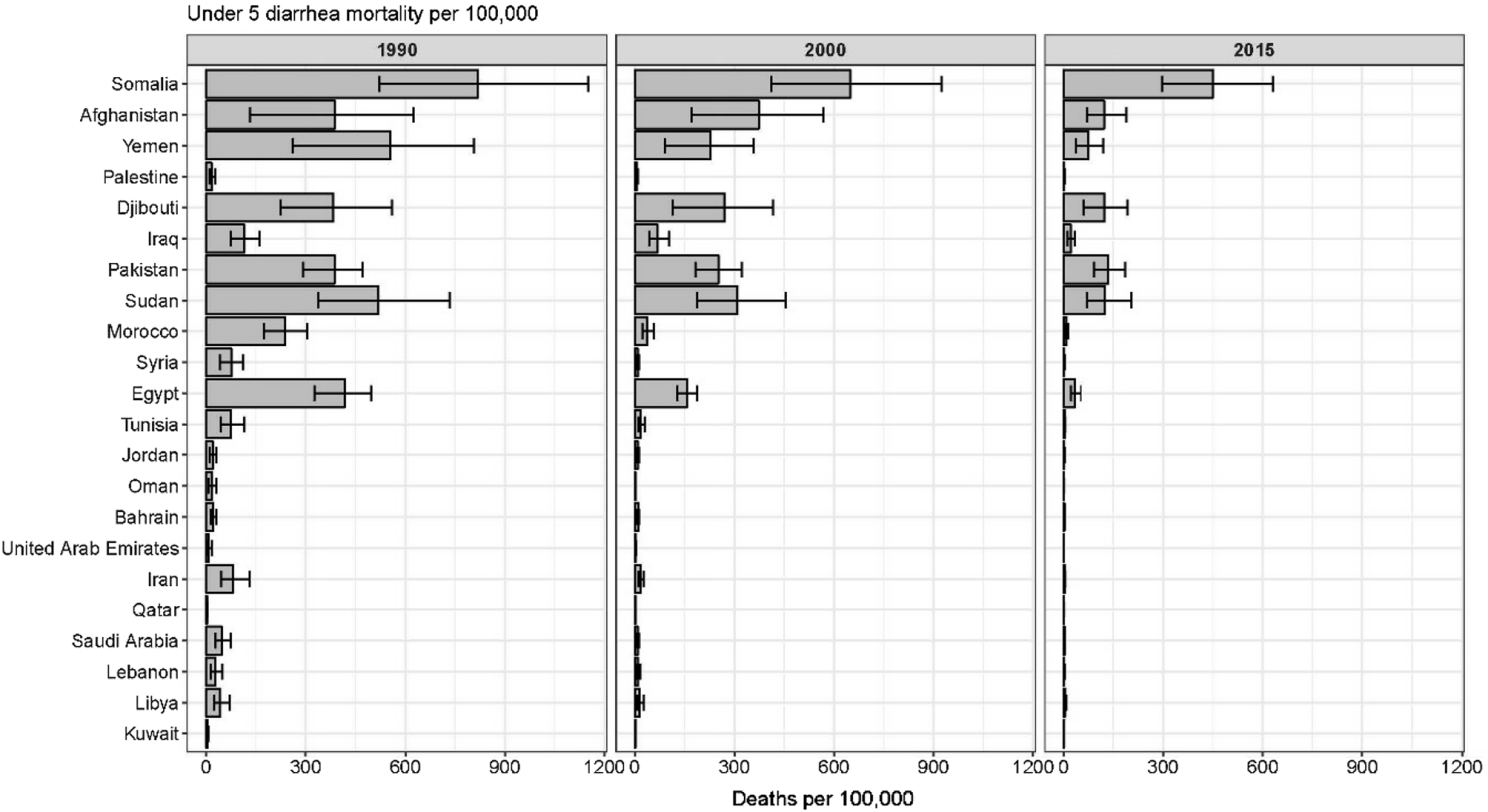
The diarrhea mortality rate per 100,000 for the Eastern Mediterranean Region by country among children under 5 in 1990, 2000, and 2015. Countries are ordered from *top* to *bottom* from lowest Socio-demographic Index (Somalia) to highest SDI (Qatar) based on SDI values in 2015. *Error bars* illustrate the 95% uncertainty interval for the estimates (Global Burden of Disease 2015 study, Eastern Mediterranean Countries, 2015)

Diarrhea mortality among children under 5 varied by time and country. The under-5 diarrhea mortality rate was highest in Somalia, followed by Pakistan, Sudan, and Afghanistan (Figs. 2, 3). Due to its high population, the greatest number of under-5 deaths occurred in Pakistan (55,500 deaths, 95% UI: 43,258–70,027). Diarrhea mortality was associated with trends in the Socio-demographic Index in most geographies (Figs. 2b, 3), but the mortality rate was much lower in Palestine and Iraq than would be expected based on SDI alone. The ratio of the observed mortality rate to the expected mortality rate based on SDI alone was lowest in Syria and Palestine, where mortality was 3% of the expected value, and highest in Bahrain, where the mortality was 65% higher than expected based on SDI alone. Syria and Palestine have moderate SDI and very low diarrhea mortality rates.

Diarrhea was also responsible for a large number of illness episodes. In 2015, diarrhea incidence was 2.1 (95% UI: 1.9–2.4) per child-year, totaling more than 171 million episodes across the EMR. The incidence was much lower in adults, including those 70+ years old (0.7 per person-year, 95% UI: 0.69–0.75). The case fatality, expressed as the number of deaths over the number of cases, was 0.1% in those 70+ years old, significantly greater than in children under 5 years (0.038%). Case fatality increased non-linearly with incidence (e-Figure 1). Despite the large observed reductions in diarrhea mortality between 1990 and 2015, diarrhea incidence decreased much more marginally (20.7%).

Among diarrheal etiologies, rotavirus was the leading cause of death in 2015 among children under 5 (13,180 deaths, 95% UI: 9807–17,738), followed by *Shigella* (10,964 deaths) and enterotoxigenic *E. coli* (ETEC) (6885 deaths) (Table 3 and e-Figure 1). Among all ages, *Shigella* was the leading cause of diarrheal deaths (19,450 deaths, 95% UI: 10,026–33,996), followed by *Aeromonas* and rotavirus. Overall, 99% of diarrheal deaths in 2015 were attributed to at least one etiology (e-Figure 2).

**Table 3:** The number of diarrhea deaths and the population attributable fraction (PAF) for 13 diarrheal etiologies among under-5 and all ages in the Eastern Mediterranean Region, 2015 (Global Burden of Disease 2015 study, Eastern Mediterranean Region, 2015)

Etiology	Under 5		All ages	
	Deaths	PAF	Deaths	PAF
Adenovirus	4392.37 (1553.83 to 9530.86)	6.69 (2.47 to 13.85)	5285.33 (1885.13 to 11,110.37)	5.1 (1.87 to 10.34)
Aeromonas	3850.51 (0 to 29,059.34)	5.83 (−35.98 to 42.15)	15,315.61 (0 to −44,627.46)	14.72 (−12.52 to 41.55)
Amoebiasis	1446.31 (0 to 12,799.8)	2.19 (−10.65 to 20.4)	3075.66 (0 to 17,604.66)	2.97 (−3.01 to 17.06)
Campylobacter enteritis	5478.32 (1421.6 to 11,408.45)	8.3 (2.35 to 16.53)	5878.95 (1504.27 to 12,465.87)	5.66 (1.51 to 11.74)
Cholera	5142.08 (2784.31 to 9223.41)	7.86 (4.27 to 14.08)	9682.46 (5614.73 to 16,600.13)	9.38 (5.37 to 16.13)
*Clostridium difficile*	8.24 (4.89 to 13.04)	0.01 (0.01 to 0.02)	12.59 (8.99 to 17.62)	0.01 (0.01 to 0.02)
Cryptosporidiosis	4569.06 (801.68 to 11,545.02)	6.92 (1.26 to 16.99)	4796.2 (699.01 to 12,482.23)	4.62 (0.71 to 11.52)
Enteropathogenic *E. coli* infection	1639.06 (132.28 to 4608.33)	2.49 (0.21 to 6.85)	1708.03 (141.29 to 4833.27)	1.64 (0.14 to 4.68)
Enterotoxigenic *E. coli* infection	6885.39 (2931.74 to 12,386.47)	10.47 (4.76 to 18.53)	11,102.89 (5152.14 to 20,035.61)	10.7 (4.92 to 18.28)
Norovirus	2402.25 (769.72 to 5439.75)	3.64 (1.26 to 8.03)	3935.54 (1027.17 to 8670.77)	3.78 (1.06 to 8.32)
Other salmonella infections	5559.16 (1663.31 to 12,323.64)	8.46 (2.57 to 18.31)	8752.77 (3002.34 to 18,462.75)	8.43 (3.01 to 17.61)
Rotaviral enteritis	13,180.3 (9807.01 to 17,738.4)	20.08 (16.37 to 25.56)	14,454.5 (10,983.35 to 18,974.79)	13.96 (11.13 to 17.68)
Shigellosis	10,963.91 (5480.23 to 19,387.69)	16.65 (9.02 to 28.57)	19,450.5 (10,025.55 to 33,996.23)	18.71 (10.32 to 30.61)

Unsafe water, sanitation, and hygiene were responsible for over 95% of diarrhea DALYs in the EMR in 2015 (95.1%, 95% UI: 89.2–98.1%), which is comparable with the global total in 2015. The proportion of diarrhea DALYs due to unsafe WASH was lowest in Jordan (87.0%) and highest in Sudan (97.0%) (e-Figure 3). Childhood undernutrition was the second-leading risk factor for diarrheal DALYs, responsible for 74.0% (95% UI: 67.4–79.1%), which is significantly higher than the global total (59.8%, 95% UI: 56.2–63.0%). Suboptimal breastfeeding was responsible for 39.5% of diarrhea DALYs among children under 5 (95% UI: 27.1–52.3%). Vitamin A and zinc deficiency were responsible for less than 10% of diarrhea DALYs among children under 5 (8 and 5%, respectively) and were lower than the global average for these risk factors (12.9 and 6.5%, respectively). Childhood undernutrition PAF ranged from 41.1% in the United Arab Emirates to 86.2% in Sudan. Diarrhea standardized exposure variables (SEV), a measure of the prevalence-weighted risk of diarrhea, was highest in Sudan, Afghanistan, and Yemen and lowest in the UAE, Jordan, and Kuwait.

## Discussion

Our study is the most comprehensive assessment of diarrheal disease burden and the contributions of specific pathogens and risk factors in the EMR to date. In 2015, the estimated diarrhea-associated deaths and DALYs showed a slight decline from the GBD 2013 estimates of 125,000 and nearly 10 million, respectively. We also continue to find significant variation within the region, with LICs and MICs experiencing social unrest bearing the vast majority of diarrheal burden. Our data clearly illustrate the gross health inequity in the region: the HICs experienced a nominal diarrhea burden compared to the substantial burden in all LICs and some MICs.

There were no major methodological changes in diarrhea mortality modeling between GBD 2013 and GBD 2015 but there were updates to the cause of death and non-fatal data. A major difference between GBD 2013 and 2015 was the introduction of the molecular diagnostic case definition for diarrheal etiologies (Liu et al. 2014; GBD 2015 Mortality and Causes of Death Collaborators 2016; GBD 2015 Risk Factors Collaborators 2016; Kassebaum et al. 2016). In the current round of GBD, we used a systematic reanalysis of the Global Enteric Multicenter Study (GEMS) that retested roughly half of the original 22,000 moderate-to-severe stool samples with quantitative polymerase chain reaction (qPCR) (Liu et al. 2016). In transitioning to a molecular diagnostic case definition, we attribute a much larger proportion of diarrhea cases and deaths to etiologies compared to previous rounds of GBD that used non-molecular diagnostics.

The Socio-demographic Index was designed to be a measure of the socio-demographic status of a country and is well correlated with the Human Development Index (GBD 2015 Mortality and Causes of Death Collaborators 2016). Still, dramatic changes in development such as those that have occurred since the start of political unrest in some of the EMR countries, such as the Syrian civil war, may be missed or at least result in a lag in the components that make up the SDI including income, fertility, and education. We have previously shown that the SDI is a good marker of disease burden, but it may not entirely capture major sociopolitical upheavals (GBD 2015 Mortality and Causes of Death Collaborators 2016).

The introduction of vaccines against diarrheal pathogens may exacerbate inequalities in diarrhea burden. For example, although rotavirus infection was the largest contributor to the diarrheal burden of disease, in some countries in the region, rotavirus vaccine is only available in the private market. This means that wealthier families, who have less need for the vaccine, will gain the primary benefit from its availability. This is troubling because economic analyses of rotavirus vaccine introduction among a number of EMR countries have uniformly suggested that vaccine introduction would be cost-beneficial from a societal perspective (Ortega et al. 2009; Connolly et al. 2012; Javanbakht et al. 2015). One study in Somalia (the only LIC country) suggested that introduction of rotavirus as a special immunization program during a complex humanitarian emergency would meet WHO cost-effectiveness benchmarks (Gargano et al. 2015).

One unique contribution of this analysis is the inclusion of all age groups. Due to the high disease burden in young children, nearly all diarrhea interventions and most diarrhea burden studies are limited to those under 5 years of age (Boschi-Pinto et al. 2008; Fischer Walker et al. 2012; Walker et al. 2013). However, the burden among those over age 70 is substantial, with diarrheal disease-associated deaths totaling nearly one sixth of the number among those under 5. The elderly may face increased diarrhea risk due to immunosenescence and comorbidities, which may also necessitate special consideration in their treatment (Trinh and Prabhakar 2007). The increasing cholera burden in five EMR countries (Jordan, Palestine, Syria, Bahrain, and Oman) is a cause for concern, as neighboring countries remain at high risk of transmission due to the presence and movement of refugee populations among them.

We found no systematic difference in under-5 diarrhea deaths or DALYs when comparing females to males. A hypothesis that there may exists evidence of differential diarrhea mortality burden by sex is supported by a previous analysis of global demographic and health survey (DHS) data. This analysis suggested that reported that girls 1–4 years old, particularly in the Middle Eastern crescent, are at a mortality disadvantage compared to boys (Hill and Upchurch 1995). This could be explained by differences in health care access and nutritional status. Furthermore, an Egyptian study found some evidence that, even when parents sought care for their daughters with diarrhea, regional health care providers provided biased treatment in favor of boys (Yount 2003). In contrast, other studies, such as a 2009 verbal autopsy study in Iraq, found no difference in under-5 mortality by sex (Awqati et al. 2009).

### Public health consequences of emergencies in the region

The EMR is facing numerous health challenges as a result of previous wars, and recent revolutions and the wars that followed (Mokdad et al. 2016; Charara et al. 2017) resulted in a huge refugee problem with millions of refugees and massive consequences for the health and well-being of millions of displaced people. The EMR now carries the largest burden of displaced populations globally.

Out of a total of 50 million refugees and internally displaced persons (IDPs) worldwide, more than 29 million (9 million refugees and 20 million IDPs) came from the region (WHO EMRO 2015). Syria is currently the world’s largest source of refugees and IDPs, with more than 40% of the population now displaced both inside the country and in neighboring states. Afghanistan and Somalia face two of the longest-spanning refugee situations, with Afghanis constituting the second-largest refugee group in the world, and Somalia facing one of the world’s most complex refugee situations (WHO EMRO 2015). Over the past few years, the region saw massive internal displacement in Iraq, with more than 3 million people fleeing their homes since June 2014, and in Yemen, where more than 2.3 million people have been internally displaced since March 2015 (ACAPS 2015).

The impact of these emergencies on public health is profound and enduring, affecting both the displaced populations and host communities. The risk of DD is increased in these settings due to limited access to safe water, rotavirus vaccines, and oral rehydration salts, along with other factors like infectious diseases, acute malnutrition, and inappropriate infant and young child feeding. The lack of safe drinking water as well as adequate sanitation and hygiene is especially concerning as a major risk factor for DD. Provision of a safe water supply, sanitation, and hygiene is a crucial priority, even in these emergency situations.

### Study limitations

Our estimates of diarrhea mortality, morbidity, and etiology attribution are limited by data availability, and although our modeling process seeks to make use of all available data, the number of relevant publications in the region is limited and unbalanced between countries. The hierarchical modeling approach allows us to “borrow” strength across time and geography to generate the best possible estimates. Second, since we only account for the acute phase of diarrhea in our YLD estimates, the resulting DALYs severely underestimate diarrhea-associated morbidity. In future GBD updates, we expect to include long-term sequelae such as stunting and cognitive impairment, (Moore et al. 2010; MacIntyre et al. 2014; Colombara et al. 2016) which will better estimate the true burden of disease.

Despite these limitations, this analysis also has several strengths. GBD methodology ensures internal consistency so that morbidity and mortality cannot be simultaneously ascribed to competing causes and allows for comparability between countries and across regions.

## Conclusion

Increased momentum of public health efforts is needed to reduce the burden of diarrhea in the EMR, especially in lower-income countries and countries experiencing political and social unrest.

Health inequities revealed in our study show that a coordinated approach that involves prevention and treatment is needed to address the multiple causes of diarrheal diseases. Regional health systems need to be strengthened to achieve the widespread availability and use of oral rehydration salts, improved nutrition, better sanitation and hygiene, and increased coverage of rotavirus immunization. Improved rates of breastfeeding should be strongly emphasized as one of the effective tools to prevent DD in countries’ public health programs.

Due to the high relevance of rotavirus infections in the EMR (Malek et al. 2010), there is also an urgent need to accelerate the rollout of rotavirus vaccine in the region through government immunization programs that would ensure access for the children who are most in need. In addition, regional governments should begin deliberation to integrate *Shigella*, ETEC, and other diarrheal vaccines that are currently in preclinical and clinical trials into their Expanded Programs on Immunization (EPI) as soon as they are approved and licensed.

## Electronic supplementary material

Below is the link to the electronic supplementary material.

Supplementary material 1 (XLSX 24 kb)

Supplementary material 2 (DOCX 582 kb)
