# Supplementary material for: Burden of diarrhea in the Eastern Mediterranean Region, 1990–2015: Findings from the Global Burden of Disease 2015 study
Source: Int J Public Health. 2017 Aug 3;63(Suppl 1):109–21. doi: 10.1007/s00038-017-1008-z (PMC5973974; doi:10.1007/s00038-017-1008-z)

Electronic Supplementary Material

**Article title:**

Burden of diarrhea in the Eastern Mediterranean Region, 1990–2015: Findings from the Global Burden of Disease 2015 study

**Journal:**

International Journal of Public Health

**Authors:**

GBD 2015 Eastern Mediterranean Region Diarrhea Collaborators

**Corresponding author:**

Ali H. Mokdad

Institute for Health Metrics and Evaluation, University of Washington, Seattle, WA, United States

Email: [mokdaa@uw.edu](mailto:mokdaa@uw.edu)

**e-Figure 1 - Etiologic distribution of diarrhea deaths in the Eastern Mediterranean Region in 2015.** The percent of diarrheal deaths by etiology among children under 5 in 2015 in the Eastern Mediterranean Region is shown. Colors and text numbers represent the population attributable fraction for each etiology by country. Etiologies are ordered from left to right by increasing number of under-5 diarrheal deaths. (Global Burden of Disease 2015 Study, Eastern Mediterranean Countries, 2015).


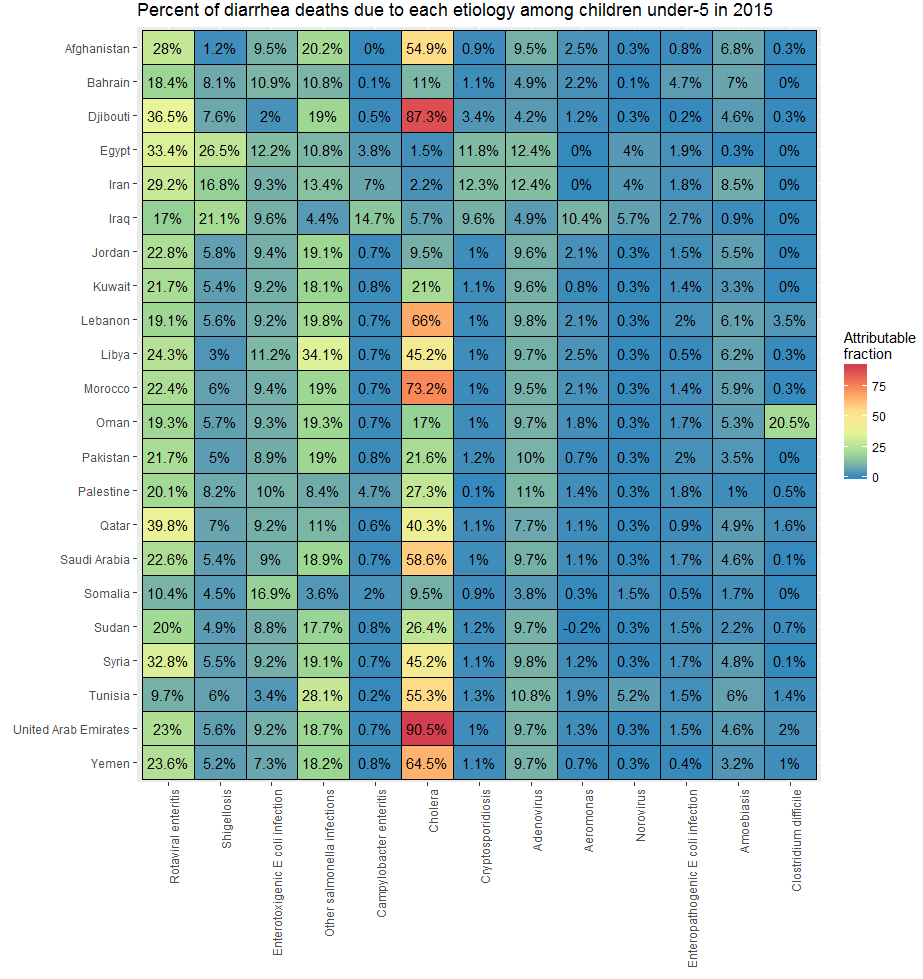


**e-Figure 2 - The distribution of diarrheal deaths attributed to each etiology for under-5 deaths (A) and for all ages (B) in the Eastern Mediterranean in 2015**. Unattributed is not estimated directly; it is the difference of 1 minus the sum of the PAFs for the diarrheal etiologies. (Global Burden of Disease 2015 Study, Eastern Mediterranean Region, 2015).

**A**


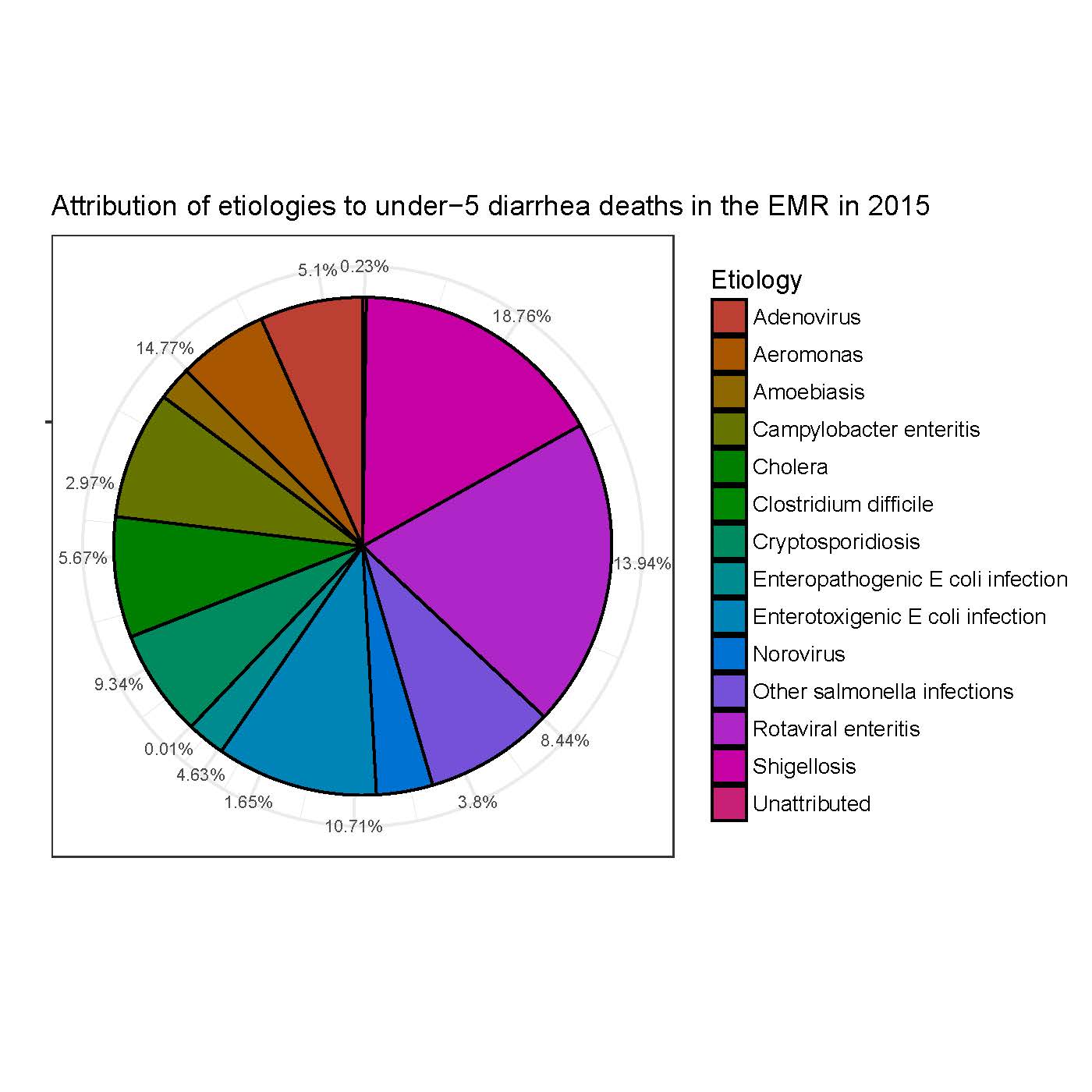


**B**


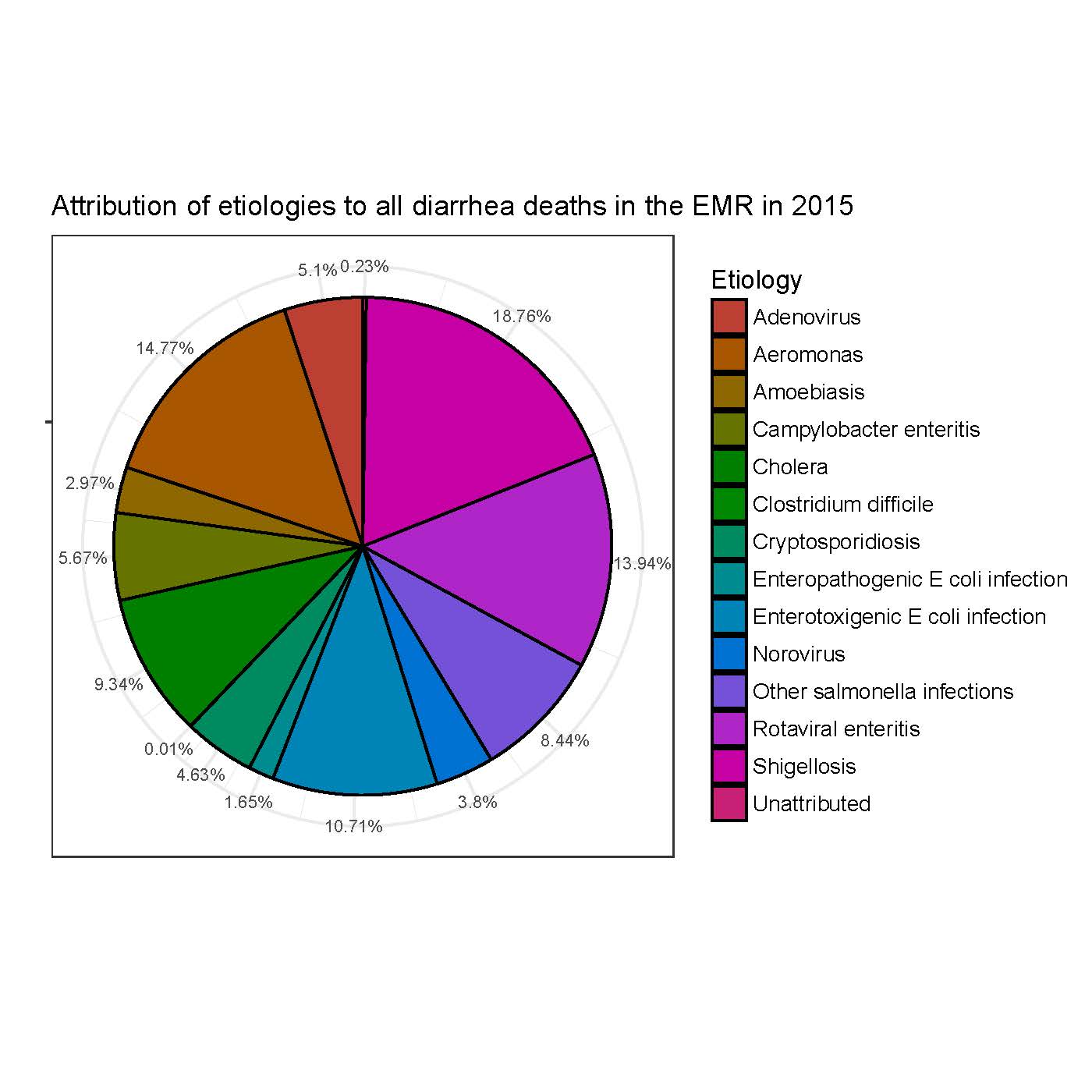


**e-Figure 3 - Population attributable fraction of risk factors to diarrhea deaths among all ages in the Eastern Mediterranean Region in 2015.** Colors and numbered text represent the attributable fraction due to each risk factor. Countries are ordered from lowest (Somalia) to highest (Kuwait). (Global Burden of Disease 2015 Study, Eastern Mediterranean Countries, 2015).


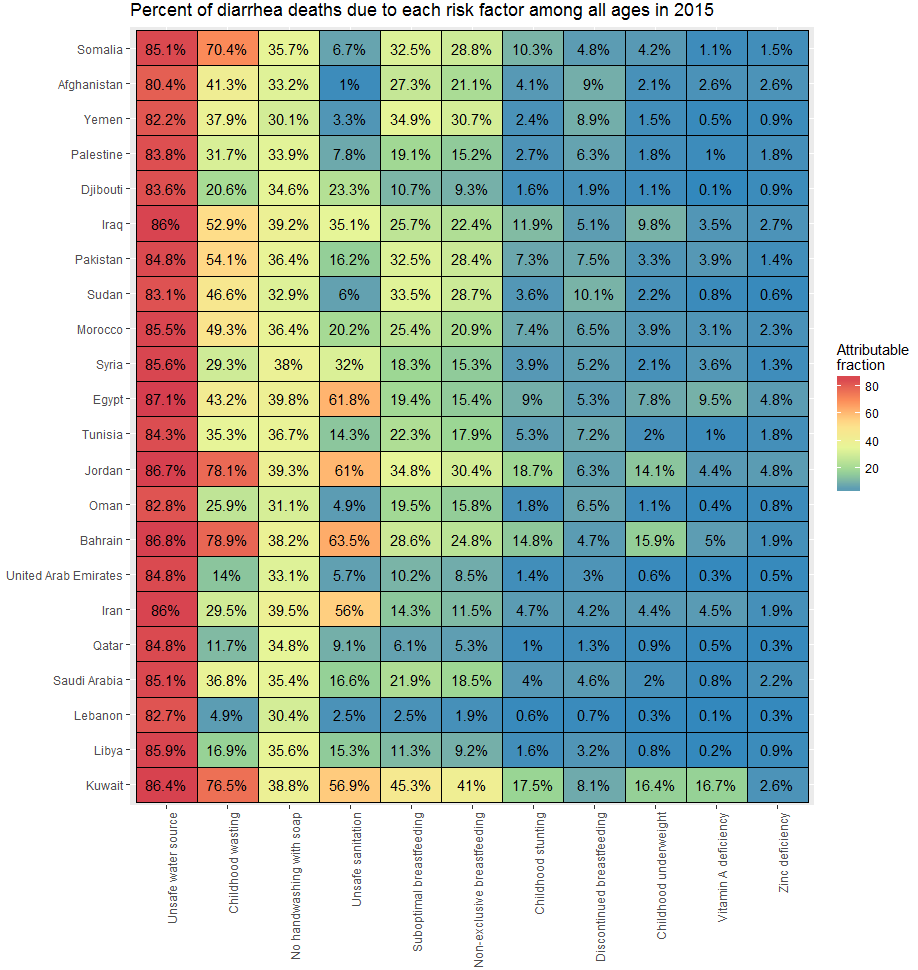

Supplement: Supplementary file 2 — Supplementary material 2 (DOCX 582 kb) [file 38_2017_1008_MOESM2_ESM.docx]
